# Flavonoid Composition and Antibacterial Properties of *Crocus sativus* L. Petal Extracts

**DOI:** 10.3390/molecules28010186

**Published:** 2022-12-26

**Authors:** Nadia Naim, Aziz Bouymajane, Yassine Oulad El Majdoub, Said Ezrari, Rachid Lahlali, Abdessalem Tahiri, Said Ennahli, Roberto Laganà Vinci, Francesco Cacciola, Luigi Mondello, Ilham Madani

**Affiliations:** 1 Department of Arboriculture-Viticulture, Ecole Nationale d’Agriculture de Meknès, Km10, Rte Haj Kaddour, BP S/40, Meknes 50001, Morocco; 2 Faculty of Sciences, Moulay Ismail University, Meknes 50001, Morocco; 3 Team of Microbiology and Health, Laboratory of Chemistry-Biology Applied to the Environment, Faculty of Sciences, Moulay Ismail University, BP 11201, Meknes 50070, Morocco; 4 Department of Chemical, Biological, Pharmaceutical and Environmental Sciences, University of Messina, 98168 Messina, Italy; 5 Department of Biology, The Faculty of Sciences and Technologies Fez, Sidi Mohamed ben Abdellah University, P.O. Box 2202, Fez 30000, Morocco; 6 Phytopathology Unit, Department of Plant Protection, Ecole Nationale d’Agriculture de Meknès, Km10, Rte Haj Kaddour, BP S/40, Menkes 50001, Morocco; 7 Department of Biomedical, Dental, Morphological and Functional Imaging Sciences, University of Messina, 98125 Messina, Italy; 8 Chromaleont s.r.l., c/o Department of Chemical, Biological, Pharmaceutical and Environmental Sciences, University of Messina, 98168 Messina, Italy; 9 Department of Sciences and Technologies for Human and Environment, University Campus Bio-Medico of Rome, 00128 Rome, Italy

**Keywords:** *Crocus sativus* L. by-product, flavonoid composition, HPLC-PDA-ESI/MS, antibacterial activity

## Abstract

Saffron petals, which are the main by-products of *Crocus sativus L.* (Iridaceae family), are produced in large quantities and are known for their many beneficial properties. In this regard, this study aims to investigate the phenolic composition and antibacterial properties of hydroethanolic extracts from *Crocus sativus* L. petals collected from Serghina (province of Boulmane) in Morocco. The phenolic profiles were characterized using high-performance liquid chromatography coupled to a photodiode array and electrospray ionization mass spectrometry (HPLC-PDA-ESI/MS). The antibacterial potential was evaluated against four bacterial strains potentially causing food-borne disease (*Staphylococcus aureus*, *Salmonella typhimurium*, *Escherichia coli*, and *Listeria monocytogenes*) using disc diffusion and broth micro-dilution assays. Results showed that a total of 27 phenolic compounds was detected in the *Crocus sativus* L. petal extracts, which were assigned to flavonoids (kaempferol, quercetin, isorhamnetin, and myricetin derivatives). The most abundant compound was represented by kaempferol-sophoroside isomer (20.82 mg/g ± 0.152), followed by kaempferol-sophoroside-hexoside (2.63 mg/g ± 0.001). The hydroethanolic extracts of *Crocus sativus* L. petals demonstrated bactericidal effects against *Staphylococcus aureus* and *Listeria monocetogenes* and bacteriostatic effects against *Escherichia coli* and *Salmonella typhimurium*. Therefore, the by-product *Crocus sativus* L. petal extracts might be considered as valuable sources of natural antibacterial agents with potential applications in the food and pharmaceutical industries.

## 1. Introduction

*Crocus sativus* L. is a perennial plant belonging to the Iridaceae family [1]. It is mainly cultivated in Iran, India, Morocco, Greece, Spain, and Italy [2]. In Morocco, saffron production has increased in recent years [3,4]. The area under cultivation was tripled (from 610 ha in 2008 to 1944 ha in 2020), and the production increased by 6.2 tons in 2020 versus only 1.5 tons in 2008 and 3.2 kg/ha instead of 2.5 kg/ha. It is widely used as a spice and as a coloring and flavoring agent in the preparation of various foods, cosmetics preparation, and diseases treatment. Saffron is known for its pharmacological properties, such as antispasmodic, expectorant, stomachache treatment, antibacterial, antiseptic, and antifungal [5,6]; antioxidant [7]; anti-inflammatory [8]; antihypertensive and hypolipidemic [9]; antidepressant [10]; and antitumor [11]. The antimicrobial activities of saffron extracts have been reported to be due to safranal and crocin compounds [12]. These compounds can easily reach the contaminant micro-organism because of their volatility and/or water solubility and contribute to microbial killing [13]. *Crocus sativus* L. is an angiosperm with a flower containing six petals, one style, three stamens, and three red–orange stigmas. In addition, saffron petals as by-products are produced in large quantities (350 kg of petals/kg of saffron stigmas), but in general, they are not used as herbal tea or food components and are thrown away after harvest [14], except in some areas where they are provided with food to flocks of domestic animals [15]. *Crocus sativus* L. petals exhibit various pharmacological effects, including antioxidant [7], antibacterial [16], anti-inflammatory, anti-diabetic, antidepressant, anti-cancer [17], anti-dyslipidemia, anti-spasmodic anti-hypertensive, and hepatoprotective properties [14,17,18,19,20]. Diverse compounds have been identified in *Crocus sativus* L. petals such as flavonoids, anthocyanins, vitamins (riboflavin and thiamine), proteins, mineral matter, and gums [19]. Previous toxicological studies have shown the toxicity of stigmas and petals at LD_50_ values of 1.6 g/kg and 6 g/kg, respectively [20]. On the other hand, *Crocus sativus* L. consumption is non-toxic to humans at 1.5 g/day, but toxic and lethal at 5 g/day, and 20 g/day, respectively [21].

Many natural compounds found in herbs and spices possess antimicrobial functions and could serve as a source of antimicrobial agents against food-borne pathogens [22]. Food poisoning is usually caused by bacterial factors and is assumed to be an acute disease followed by eating contaminated food or beverages [23]. Numerous studies have highlighted the antimicrobial properties of natural plant extracts, basically due to their richness with different classes of phenolic compounds [24]. Antibiotics have been used for the treatment of infectious diseases for a long time. However, antimicrobial resistance, among pathogenic bacteria, against drugs used in treating human infection is increasing. This situation has forced scientists to search for new antimicrobial substances from various plants, which are good sources of novel antimicrobial chemotherapeutic agents [25]. Because of negative consumer perceptions of chemical preservatives, attention is shifting towards natural alternatives. Therefore, the aim of the present study was to investigate the phenolic compositions and to evaluate the antibacterial activities of hydroethanolic extracts from *Crocus sativus* L. petals collected from a newly cultivated mountain region in Morocco (Serghina/Boulmane).

## 2. Results

### 2.1. HPLC-PDA-ESI/MS Analysis

**Figure 1:**
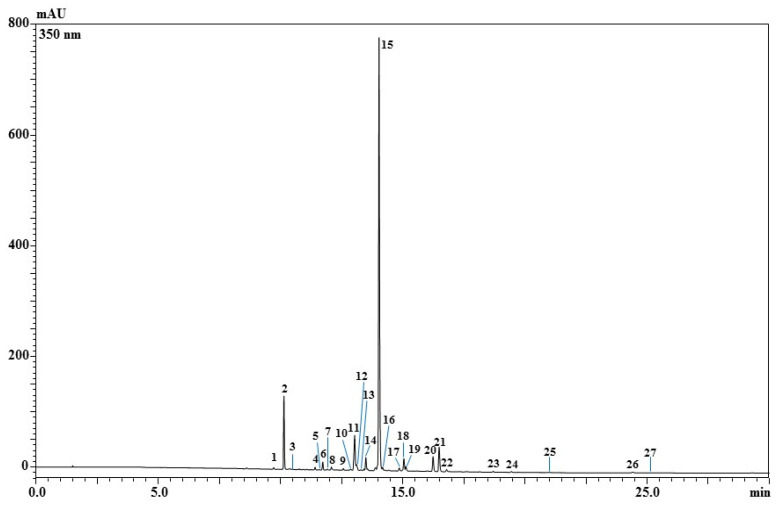
Phenolic compound analysis by HPLC-PDA-ESI-MS of saffron petal extract (EtOH:H_2_O 80:20 *v*/*v*) acquired at 350 nm.

Phenolic profile analyses were carried out using high-performance liquid chromatography coupled to a photodiode array and electrospray ionization mass spectrometry (HPLC-PDA/ESI-MS) (Figure 1). As listed in Table 1, a total of twenty-seven phenolic compounds was detected according to standards, λ_max_, retention times, mass spectrometry, and literature data. Compounds were assigned to flavonoids (kaempferol, quercetin, isorhamnetin, and myricetin derivatives). In terms of quantification, the kaempferol-sophoroside isomer turned out to be the most abundant one in the saffron petal extract (20.82 mg/g ± 0.152), followed by kaempferol-sophoroside-hexoside (2.63 mg/g ± 0.001).

### 2.2. Antibacterial Activity

The antibacterial activity of *Crocus sativus* L. petal hydroethanolic extracts was evaluated against Gram-positive bacteria (*Listeria monocytogenes* and *Staphylococcus aureus*) and Gram-negative bacteria (*Escherichia coli* and *Salmonella typhimurium*) by agar diffusion and broth microdilution assays. As indicated in Table 2, the hydroethanolic extracts of *Crocus sativus* L. petals revealed significant antibacterial activity against all tested bacteria, with inhibition zone diameters ranging from 7 to 15 mm. The extracts showed maximum zone inhibition against *Listeria monocytogenes* (15 mm), followed by *Salmonella typhimurium* (12 mm), *Staphylococcus aureus* (9 mm), and *Escherichia coli* (7 mm).

The minimum inhibitory concentration (MIC) of *Crocus sativus* L. petal hydroethanolic extracts was tested against four pathogenic bacteria. As shown in Table 2, all the tested bacteria were sensitive to amoxicillin, with MIC values ranging from 15.62 ± 0.00 to 31.28 ± 0.00 µg/mL, while the MIC values for hydroethanolic extracts against all bacteria tested ranged from 4.33 ± 1.50 to 6.936 ± 3.01 mg/mL. Thus, the hydroethanolic extracts showed the most effective MIC values for all bacteria tested. The minimum bactericidal concentrations (MBC) of hydroethanolic extracts ranged between 13.88 ± 6.01 and 41.66 ± 0.00 mg/mL (Table 2). Based on the MBC/MIC ratio, the hydroethanolic extracts showed bacteriostatic effects against *Escherichia coli* and *Salmonella typhimurium* and bactericidal effects against *Staphylococcus aureus* and *Listeria monocytogenes*.

## 3. Discussion

The treatment of infectious diseases is mainly achieved by applying antibiotics; however, antimicrobial resistance, among pathogenic bacteria, against drugs used in treating human infection is increasing. This situation has forced scientists to search for new antimicrobial substances from various plants, which are good sources of novel antimicrobial chemotherapeutic agents [25]. In this study, a saffron petal extract was investigated for its phenolic profile and antibacterial activities as a potential alternative strategy to substitute the use of chemicals. *Crocus sativus* L. petal is the main by-product of *Crocus sativus* L. that is produced in large quantities and is known for several properties such as antibacterial potential.

In this study, phenolic profile analysis was carried out using high-performance liquid chromatography coupled to a photodiode array and electrospray ionization mass spectrometry (HPLC-PDA/ESI-MS). The obtained results showed that the major compounds were assigned to flavonoids (kaempferol derivatives, quercetin derivatives, isorhamnetin derivatives, and derivatives of myricetin). As has already been reported in previous studies, the primary classes of phenolic compounds in *Crocus sativus* L. petals are flavonoids [29]. The *Crocus sativus* petal extract is especially rich in flavonols, particularly derivatives of kaempferol, quercetin, and isorhamnetin [29,30]. Indeed, flavonoids are very important constituents of plants because of the scavenging ability conferred by their hydroxyl groups. Moreover, Termentzi et al. [31] found that *Crocus sativus* L. petal is a good potential source of quercetin, kaempferol, and naringenin, which are relatively highly resistant flavonols to thermal degradation.

Regarding the antibacterial activity, the obtained results showed that the hydro-ethanolic extract of *Crocus sativus* L. petals indicated significant antibacterial activity against all tested bacteria. Previous studies showed that a hydroalcoholic extract of *Crocus sativus* L. petal possessed antibacterial activity against *Staphylococcus aureus*, *Bacillus cereus*, *Salmonella typhimurium*, *Escherichia coli*, and *Shingella dysenteriae* at a concentration of 1000 mg/mL, with inhibition zone diameters ranging from 13 to 22 mm [32]. In addition, the hydro-ethanolic extract of *Crocus sativus* L. petals showed higher zone inhibition against *Listeria monocytogenes* than the standard antibacterial amoxicillin. According to a previous study conducted by Nasab [14], *Crocus sativus* L. petals had a higher effect than the amoxicillin antibiotic.

As shown in Table 2, the hydroethanolic extract showed the most effective MIC values for all bacteria tested. The antibacterial effect of the *Crocus sativus* L. petal extract has been evaluated in several studies. These studies showed different values for the MIC of the hydroalcoholic extract of saffron petals on the tested bacteria. In the same studies, the MIC values were higher than those reported in our study, especially for *Escherichia coli* (125 mg/mL) and *Salmonella typhimurium* (62.5 mg/mL) [32]. The obtained values revealed that the hydroethanolic extract had greater antibacterial activity than another extract; this can be explained by the fact that an ethanolic extract is the preferred solvent to extract phenol, flavonoids, and other antioxidant material of herbs with antibacterial activities [16,33]. Based on the MBC/MIC value ratio, the hydroethanolic extract showed a bacteriostatic effect against *Escherichia coli* and *Salmonella typhimurium* and a bactericidal effect against *Staphylococcus aureus and Listeria monocytogenes*. The obtained results were consistent with several previous studies [14,32,34]. It has been well confirmed that the antibacterial activity of plant extracts depends on their chemical composition and type of bacteria.

Regarding chemical composition, phenolic compounds have shown great antimicrobial activity [34]. In addition, the results obtained revealed that *Crocus sativus* L. petal hydroethanolic extract had more antibacterial activity against Gram-positive bacteria. This result is consistent with the finding of Nasab et al. [14]. According to previous findings, Gram-negative bacteria have an effective permeability barrier and are not susceptible to plant extracts compared to Gram-positive bacteria [35]. This resistance is due to the presence of phospholipids and lipopolysaccharides in the membrane of Gram-negative bacteria, which can be an effective barrier against antibacterial agents [36]. On the other hand, Gram-positive bacteria have a mesh-like peptidoglycan layer, which is more accessible to permeation by the extracts [37]. In a previous study, it was found that a hydroalcoholic extract from saffron petals had an antibacterial effect, especially on *L. monocytogenes* and *E. coli* [14]. The antibacterial activity of *Crocus sativus* L. petal extracts against *Escherichia coli, Pseudomonas aeruginosa, Klebsiella pneumonia, Shigella flexneri*, and *Bacillus subtilis* has also been reported by Alzoreky et al. and Bouymajane et al. [38,39].

The obtained results confirm the implication of phenolic compounds contained in *Crocus sativus* L. petals in the investigated antibacterial activity. Phenolic contents were highlighted as very powerful antimicrobial agents that exert a direct effect by neutralizing microbial systems and damaging the hyphae [40]. Another study showed that flavonoid-rich plant extracts from different plants possess antibacterial activity [41]. The antibacterial activity from petals of *Crocus sativus* might be due to the presence of kaempferol, quercetin, and isorhamnetin. The mechanisms of action by which plant extracts suppress the growth of microbial pathogenes are multiple and include disruption of cell membrane function, disruption of energy activity, and damage to the cytoplasmic membrane [42]. In addition, these samples have been shown to possess antioxidant activity [43]. According to Rice-Evans et al. and She et al. [44,45], the antioxidant capacity of plant extracts is closely associated with phenolic components, which could interact with the free radicals by electrons or hydrogen. The flavonoids may contribute directly to anti-oxidative and antimicrobial action [46]. Moreover, the most common causes of bacterial food-borne diseases are *Salmonella typhimurium*, *Staphylococcus aureus*, *Escherichia coli*, and *Shigella flexneri* [38]. The prevention and treatment of disease caused by these organisms are complicated by the increase in multidrug-resistant strains and the lack of an effective vaccine and preservatives. Therefore, *Crocus sativus* L. petals, which are discarded in quantities of thousands of tons each year, could be promising natural products that play the role of antioxidant sources, which could improve product quality in the cosmetic and pharmaceuticals industries and act as natural sources of antibacterial agents with industrial applications.

## 4. Materials and Methods

### 4.1. Plant Material

Saffron petals were collected in November 2021 after pruning the harvest from a saffron farm in Serghina/Boulmane (Morocco). The petals were stored at 37 °C to absorb the humidity. Saffron petals were crushed using an automated grinder, and then 10 g of the petals was homogenized in 100 mL of ethanol 80% and stirred for 24 h at room temperature. The mixture was filtered and placed in an oven to remove the solvent until obtaining a dry extract.

### 4.2. HPLC-PDA-ESI/MS Analysis

An EtOH/H_2_O extract of *Crocus sativus* L. petals was analyzed by high-performance liquid chromatography coupled to a photodiode array and electrospray ionization mass spectrometry (HPLC-PDA-ESI/MS). The identification of compounds was conducted by comparing mass spectra obtained with literature data.

#### 4.2.1. Sample Preparation

A dried extract of *Crocus sativus* petals was redissolved in the same organic solvents and diluted 1:30 (*v/v*). The mixture was filtered through a 0.2 μm Acrodisc nylon membrane (Merck Life Science, Merck KGaA, Darmstadt, Germany) prior to HPLC-PDA-ESI/MS.

#### 4.2.2. HPLC-PDA-ESI/MS analysis condition

Chromatographic analysis was accomplished by means of a Shimadzu HPLC system (Kyoto, Japan) equipped with a CBM-20A controller, two LC-20AD dual-plunger parallel-flow pumps, a DGU20A5R degasser, a CTO-20AC column oven, a SIL-30AC autosampler, an SPD-M20A photodiode array detector, and an LCMS-2020 single quadrupole mass spectrometer, with the employment of an ESI source operated in negative and positive ionization modes.

Chromatographic separations were carried out on Ascentis Express RP C18 columns (150 × 4.6 mm; 2.7 μm) (Merck Life Science, Merck KGaA, Darmstadt, Germany) [47,48,49]. The employed mobile phase was composed of two solvents: water (solvent A) and ACN (solvent B), both acidified with 0.10% formic acid *v*/*v*. The flow rate was set at 1 mL/min, under gradient elution 0–5 min, 0–5% B; 10 min, 15% B; 20 min, 30% B; 60 min, 50% B; 70 min, 100% B; 75 min, 100% B. The injection volume was 5 μL. Diode array detection (DAD) was applied in the range of 200–600 nm and monitored at a wavelength of 330 nm (sampling frequency: 12.5 Hz, time constant: 0.160 s). MS conditions were as follows: scan range and the scan speed were set at a mass-to-charge ratio (*m*/*z*) 100–1600 and 7500 amu/s, respectively; event time: 0.3 s, nebulizing gas (N_2_) flow rate: 1.5 L/min, drying gas (N_2_) flow rate: 15 L/min, interface temperature: 350 °C, heat block temperature: 300 °C, desolvation line temperature: 300 °C, desolvation line voltage: 1 V, interface voltage: −4.5 kV.

#### 4.2.3. Standards Employed

Calibration curves of three polyphenolic standards (quercetin-3-O-glucopyranoside, kaempferol-3-O-glucoside, isorhamnetin-3-O-glucoside) were employed for the quantification of the polyphenolic content in sample extracts. Each analysis was performed in 6 repetitions. Data acquisition was performed by Shimadzu Lab Solution software ver. 5.99.

### 4.3. Antibacterial Activity

#### 4.3.1. Bacterial Strains and Growth Conditions

The bacterial strains used in this study were obtained from the Laboratory of Microbiology and Health at Faculty of Sciences, Moulay Ismail University, Morocco. The antibacterial activity of *Crocus sativus* L. extract was assessed using four pathogenic bacteria (*Escherichia coli, Salmonella typhimurium, Staphylococcus aureus,* and *Listeria monocytogenes*). Bacterial strains from the frozen stock (–80 °C) were prepared by seeding on tryptone soy yeast extract agar medium (TSYEA; Biolife, Milan, Italy) and incubated at 37 °C for 24 h. Then, bacterial suspensions were prepared in sterile distilled water and adjusted to the equivalent of 0.5 McFarland standard (10^8^ CFU/mL).

#### 4.3.2. Disc Diffusion Method

The agar disc diffusion assay was performed in order to test the preliminary antimicrobial activity of extract from *Crocus sativus* L. petals according to the method proposed by Bouymajane et al. [39]. Briefly, 100 µL of each bacterial suspension (*Escherichia coli, Salmonella typhimurium, Staphylococcus aureus,* and *Listeria monocytogenes*) was spread on Petri dishes containing Mueller Hinton agar (Merck Life Science, Merck KGaA, Darmstadt, Germany). Then, sterile filter discs (diameter 6 mm, Whatman Paper No. 1) were placed on Petri dishes and impregnated with 10 µL of ethanol extract from *Crocus sativus* L. petals (1000 mg/mL). Amoxicillin (10 µg/disc) was used as a reference. Then all Petri dishes were incubated at 37 °C for 24 h, and the diameters of inhibition zones were measured in millimeters.

#### 4.3.3. Broth Microdilution Method

The minimum inhibitory concentration (MIC) and the minimum bactericidal concentration (MBC) were evaluated by the broth microdilution assay [39]. In flat-bottom 96-well microplates, 50 µL of sterile distilled water were added to each microplate well. Then, 50 µL of dried extract mixed with sterile distilled water (1000 mg/mL) was added to the first well plate and mixed in order to determine the serial dilutions. Additionally, 50 µL of tryptone soy yeast extract broth and 50 µL of bacterial suspension were added to each well. The well containing the bacterial suspension with SYEB and the well containing sterile distilled water and extract were served as positive and negative controls, respectively. Therefore, amoxicillin (30 µg/mL) was used as a reference drug according to the method described by the Clinical and Laboratory Standards Institute (CLSI 2021). After microplate incubation at 37 °C for 24 h, 40 µL of TTC (2,3,5-triphenyl tetrazolium chloride) was added to each well and reinsulated at 37 °C for 30 min.

The MIC values were determined at the lowest concentrations of extracts at which bacterial growth was not observed. At the same time, the MBC values were determined to have the lowest concentrations of extract that did not produce a bacterial colony by plating 100 µL of samples from wells in which no growth was observed on tryptone soy yeast extract agar and incubated at 37 °C for 24 h. The MBC/MIC ratio was used to determine the bacteriostatic and bactericidal effects of the extract. If the MBC/MIC ratio was less than 4, the effect was bactericidal, and if the ratio was greater than 4, the effect was bacteriostatic.

### 4.4. Statistical Analysis

All the experiments were carried out in triplicate. Statistical analysis was performed by SPSS V25 software (version 25, IBM SPSS Statistics 20, New York, NY, USA) based on the data, and the obtained results were expressed as the mean ± standard deviation. The data were then compared using Duncan’s multiple range tests at 5% significance levels.

## 5. Conclusions

In the present study, hydroethanolic extracts from *Crocus sativus* L. petals collected from the Serghina (province of Boulmane) in Morocco were characterized for their phenolic profiles and evaluated for their antibacterial properties. Results showed that the *Crocus sativus* L. petal extracts displayed great potential antibacterial properties, which might be due to the presence in its chemical composition of kaempferol, quercetin, and isorhamnetin derivatives. Therefore, it can be suggested that petals of *Crocus sativus* L. could be considered as a source of antimicrobial agents, which might be applied in pharmaceutical products.

## Figures and Tables

**Table 1:** Semi-quantification of phenolic compounds in MeOH:H_2_O (80:20 *v*/*v*) extracts of *Crocus sativus* petals detected by LC-PDA/ESI-MS analysis. Quantification of phenolic compounds was reported in mg/g of dried extract ± SD (*n* = 3).

Peak	Compound	t_r_ (min)	UV Max (nm)	[M-H]^-^	mg/g ± SD	Ref.
**1**	Myricetin-rutinoside-hexoside	9.70	255, 352	787, 463	0.08 ± 0.000	[26]
**2**	Kaempferol-sophoroside-hexoside	10.08	266, 346	771, 609, 285	2.63 ± 0.001	[26]
**3**	Kaempferol-rutinoside-hexoside	10.46	265, 344	755, 593	0.11 ± 0.003	[26]
**4**	Kaempferol-glucosyl-(6”-acetylgalactoside)-hexoside	11.37	265, 347	813, 651	0.19 ± 0.000	[26]
**5**	Eriodictyol-hexoside derivative	11.52	282, 331sh	625, 449, 287	x	-
**6**	Kaempferol-sophoroside isomer	11.68	265, 345	609, 447, 285	0.39 ± 0.008	[26]
**7**	Unknown	11.88	271, 334	787, 602, 266	x	-
**8**	Myricetin-dihexoside	12.04	260, 353	641, 479, 317	0.22 ± 0.044	-
**9**	Kaempferol (or Luteolin)-dihexoside derivative	12.53	265, 344	695, 447, 285	0.18 ± 0.008	-
**10**	Kaempferol (or Luteolin)-trihexoside derivative isomer	12.83	266, 348	873, 771, 447, 285	0.16 ± 0.042	-
**11**	Quercetin-sophoroside	12.98	254, 352	625, 463, 301	2.45 ± 0.008	[26]
**12**	Kaempferol (or Luteolin)-trihexoside derivative isomer	13.08	266, 348	873, 771, 447, 285	0.43 ± 0.001	-
**13**	Kaempferol-hexoside isomer	13.27	267, 347	447	0.12 ± 0.001	[26]
**14**	Isorhamnetin-sophoroside	13.45	252, 344	639, 477, 315	0.76 ± 0.005	[26]
**15**	Kaempferol-sophoroside isomer	14.00	265, 347	609, 447, 285	20.82 ± 0.152	[26]
**16**	Isorhamnetin-sophoroside	14.16	255, 352	639, 477, 315	0.11 ± 0.015	[26]
**17**	Kaempferol-rutinoside	14.85	265, 347	593, 285	0.25 ± 0.005	[26]
**18**	Isorhamnetin-rutinoside	15.03	254, 353	623, 477, 315	0.76 ± 0.021	[26]
**19**	Quercetin-hexoside	15.12	255, 354	463, 301	0.29 ± 0.006	[26]
**20**	Kaempferol-(6”-acetyl-glucoside)-glucoside	16.22	265, 347	651, 489, 285	0.82 ± 0.012	[26]
**21**	Kaempferol-hexoside isomer	16.47	264, 346	447, 285	1.33 ± 0.009	[26]
**22**	Isorhamnetin-hexoside	16.78	254, 356	477, 315	0.16 ± 0.002	[27]
**23**	Kaempferol (or Luteolin)-derivative	18.69	265, 347	489, 285	0.14 ± 0.001	-
**24**	Unknown	19.44	266, 311, 352 sh	609	x	-
**25**	Unknown	21.01	266, 313, 355 sh	593	x	-
**26**	Kaempferol	24.41	264, 366	285	<LOQ	[28] Standard
**27**	Isorhamnetin	25.15	371	315	<LOQ	Standard

x: detected but not quantified; sh: wavelength shoulder.

**Table 2:** Determination of the diameter of the inhibition zone (D, mm), the minimum inhibitory concentration (MIC), and the minimum bactericidal concentration (MBC) of amoxicillin and hydroethanolic extract obtained from the *Crocus sativus* L. petals (mg/mL).

Bacteria	Amoxicillin				*Crocus sativus* L. extract			
	D	MIC	MBC	MBC/MIC	D	MIC	MBC	MBC/MIC
*Escherichia coli*	8 ± 0.12 ^a^	0.015 ± 0.00 ^a^	0.012 ± 0.00 ^a^	1	7 ± 0.10 ^a^	4.33 ± 1.50 ^a^	34.72 ± 12.02 ^b^	8
*Salmonella typhimurium*	27 ± 0.22 ^c^	0.015 ± 0.00 ^a^	0.012 ± 0.00 ^a^	1	12 ± 0.06 ^b^	6.94 ± 3.01 ^b^	41.66 ± 0.00 ^b^	6
*Staphylococcus aureus*	30 ± 0.33 ^c^	0.015 ± 0.00 ^a^	0.02 ± 0.00 ^a^	1	9 ± 0.14 ^a^	6.94 ± 3.007 ^b^	13.88 ± 6.01 ^a^	2
*Listeria monocytogenes*	12 ± 0.04 ^b^	0.031 ± 0.00 ^a^	0.03 ± 0.00 ^a^	1	15 ± 0.03 ^b^	4.33 ± 1.50 ^a^	17.35 ± 6.01 ^a^	4

Values followed by different letters (a–c) in the same column are significantly different according to Duncan’s test (*p* < 0.05).

## Data Availability

Not applicable.

## Sample Availability

Samples of the compounds are available from the authors.
